# Real-world evidence of biologic treatments in psoriatic arthritis in Italy: results of the CHRONOS (EffeCtiveness of biologic treatments for psoriatic artHRitis in Italy: an ObservatioNal lOngitudinal Study of real-life clinical practice) observational longitudinal study

**DOI:** 10.1186/s41927-022-00284-w

**Published:** 2022-09-12

**Authors:** Delia Colombo, Micol Frassi, Giusy Pagano Mariano, Enrico Fusaro, Claudia Lomater, Patrizia Del Medico, Florenzo Iannone, Rosario Foti, Massimiliano Limonta, Antonio Marchesoni, Bernd Raffeiner, Ombretta Viapiana, Walter Grassi, Rosa Daniela Grembiale, Giuliana Guggino, Antonino Mazzone, Enrico Tirri, Roberto Perricone, Pier Carlo Sarzi Puttini, Salvatore De Vita, Fabrizio Conti, Alessandra Ori, Lucia Simoni, Martina Fiocchi, Roberto Orsenigo, Emanuela Zagni, and the CHRONOS Study Group, Micol Frassi, Maurizio Caminiti, Enrico Fusaro, Claudia Lomater, Patrizia Del Medico, Florenzo Iannone, Rosario Foti, Massimiliano Limonta, Antonio Marchesoni, Bernd Raffeiner, Ombretta Viapiana, Walter Grassi, Rosa Daniela Grembiale, Giuliana Guggino, Antonino Mazzone, Enrico Tirri, Roberto Perricone, Pier Carlo Sarzi Puttini, Salvatore De Vita, Fabrizio Conti

**Affiliations:** 1 Novartis Farma S.p.A, Largo Umberto Boccioni, 1, 21040 Origgio, Varese Italy; 2 ASST Spedali Civili, Brescia, Italy; 3 Grande Ospedale Metropolitano Bianchi Melacrino Morelli, Reggio Calabria, Italy; 4 AOU Città della Salute e della Scienza di Torino, Turin, Italy; 5 A.O. Mauriziano, Turin, Italy; 6 Ospedale Civile, Civitanova Marche, Italy; 7 A.O.U. Policlinico Consorziale, Bari, Italy; 8 A.O.U. Policlinico -Vittorio Emanuele, Catania, Italy; 9 ASST Papa Giovanni XXIII, Bergamo, Italy; 10 Department of Rheumatology, ASST Gaetano Pini-CTO, Milan, Italy; 11 Ospedale Centrale di Bolzano, Bolzano, Italy; 12 AOUI Verona Borgo Rome, Verona, Italy; 13 Policlinico A. Murri, Jesi, Italy; 14 A.O.U. Mater Domini, Catanzaro, Italy; 15 A.O.U. Policlinico Giaccone, Palermo, Italy; 16 Ospedale Civile Di Legnano, Legnano, Italy; 17 Ospedale San Giovanni Bosco, Naples, Italy; 18 Policlinico Tor Vergata, Rome, Italy; 19 ASST FBF Sacco, Milan, Italy; 20 ASUIUD, Udine, Italy; 21 Azienda Policlinico Umberto I, Rome, Italy; 22 MediNeos Observational Research, Modena, Italy; 23 Rheumatology, Humanitas San Pio X, Milan, Italy

**Keywords:** Psoriatic arthritis, Biologics, Real world evidence, ACR, DAS28, Secukinumab, TNF-inhibitors

## Abstract

**Background:**

Biologics have demonstrated efficacy in PsA in randomized clinical trials. More evidence is needed on their effectiveness under real clinical practice conditions. The aim of the present work is to provide real-world evidence of the effectiveness of biologics for PsA in the daily clinical practice.

**Methods:**

CHRONOS was a multicenter, non-interventional, cohort study conducted in 20 Italian hospital rheumatology clinics.

**Results:**

399 patients were eligible (56.9% females, mean (SD) age: 52.4 (11.6) years). The mean (SD) duration of PsA and psoriasis was 7.2 (6.9) and 15.3 (12.2) years, respectively. The mean (SD) duration of the biologic treatment under analysis was 18.6 (6.5) months. The most frequently prescribed biologic was secukinumab (40.4%), followed by adalimumab (17.8%) and etanercept (16.5%). The proportion of overall responders according to EULAR DAS28 criteria was 71.8% (95% CI: 66.7–76.8%) out of 308 patients at 6 months and 68.0% (95% CI: 62.7–73.3%) out of 297 patients at 1 year. Overall, ACR20/50/70 responses at 6 months were 41.2% (80/194), 29.4% (57/194), 17.1% (34/199) and at 1-year were 34.9% (66/189), 26.7% (51/191), 18.4% (36/196), respectively. Secondary outcome measures improved rapidly already at 6 months: mean (SD) PASI, available for 87 patients, decreased from 3.2 (5.1) to 0.6 (1.3), the proportion of patients with dactylitis from 23.6% (35/148) to 3.5% (5/142) and those with enthesitis from 33.3% (49/147) to 9.0% (12/133).

**Conclusions:**

The CHRONOS study provides real-world evidence of the effectiveness of biologics in PsA in the Italian rheumatological practice, confirming the efficacy reported in RCTs across various outcome measures.

**Supplementary Information:**

The online version contains supplementary material available at 10.1186/s41927-022-00284-w.

## Background

Psoriatic disease has been recognized as a systemic inflammatory disorder affecting the skin, the nails and the joints, and that can be complicated by systemic comorbidities [1, 2]. Psoriatic arthritis (PsA) is a seronegative spondyloarthropathy characterized by musculoskeletal signs and symptoms (arthritis, enthesitis, dactylitis, axial disease) with associated pain and tenderness in the involved sites [3, 4]. In the majority of patients, the skin symptoms of psoriasis develop first, followed by the arthritis; however, in 15% of cases, arthritis precedes the skin manifestations [5]. In Italy, PsA affects an estimated 0.3–1.0% of the general population [6]. Globally, it accounts for around 20% of referrals to the early arthritis clinic and its prevalence in patients with psoriasis is estimated around 30% (ranging from 18 to 42%, depending on geographic region) [7]. Early diagnosis is crucial to ensure optimal management and prevent long-term functional disability.

Improved understanding of the pathogenesis of PsA has led to the development of biologic medications and small molecules targeting specific cytokines and signaling pathways, which have shown to prevent disease progression and improve quality of life. Almost two decades ago, tumor necrosis factor inhibitors (TNFis) were the first biologic disease modifying anti-rheumatic drugs (DMARDs) approved for the treatment of PsA and since then several new biologic agents have been developed, targeting interleukin (IL) 12/23, IL-23, and IL17 [5, 8, 9]. These biologic agents are recommended for the treatment of active moderate-severe PsA in adults with inadequate response to previous non-biologic DMARDs [10]. Currently, the biologic medications that have demonstrated efficacy in PsA in randomized clinical trials (RCTs), and have been commercialized in Italy, include TNFi, the IL12/23 inhibitor ustekinumab, the IL17A inhibitors secukinumab and ixekizumab [11–16]. However, more evidence is needed on the effectiveness of these agents under real clinical practice conditions. The present study was designed to provide real-world evidence (RWE) of the effectiveness of biologic treatments for PsA in the Italian real-life clinical practice.

## Methods

### Study design and participants

**Fig. 1:**
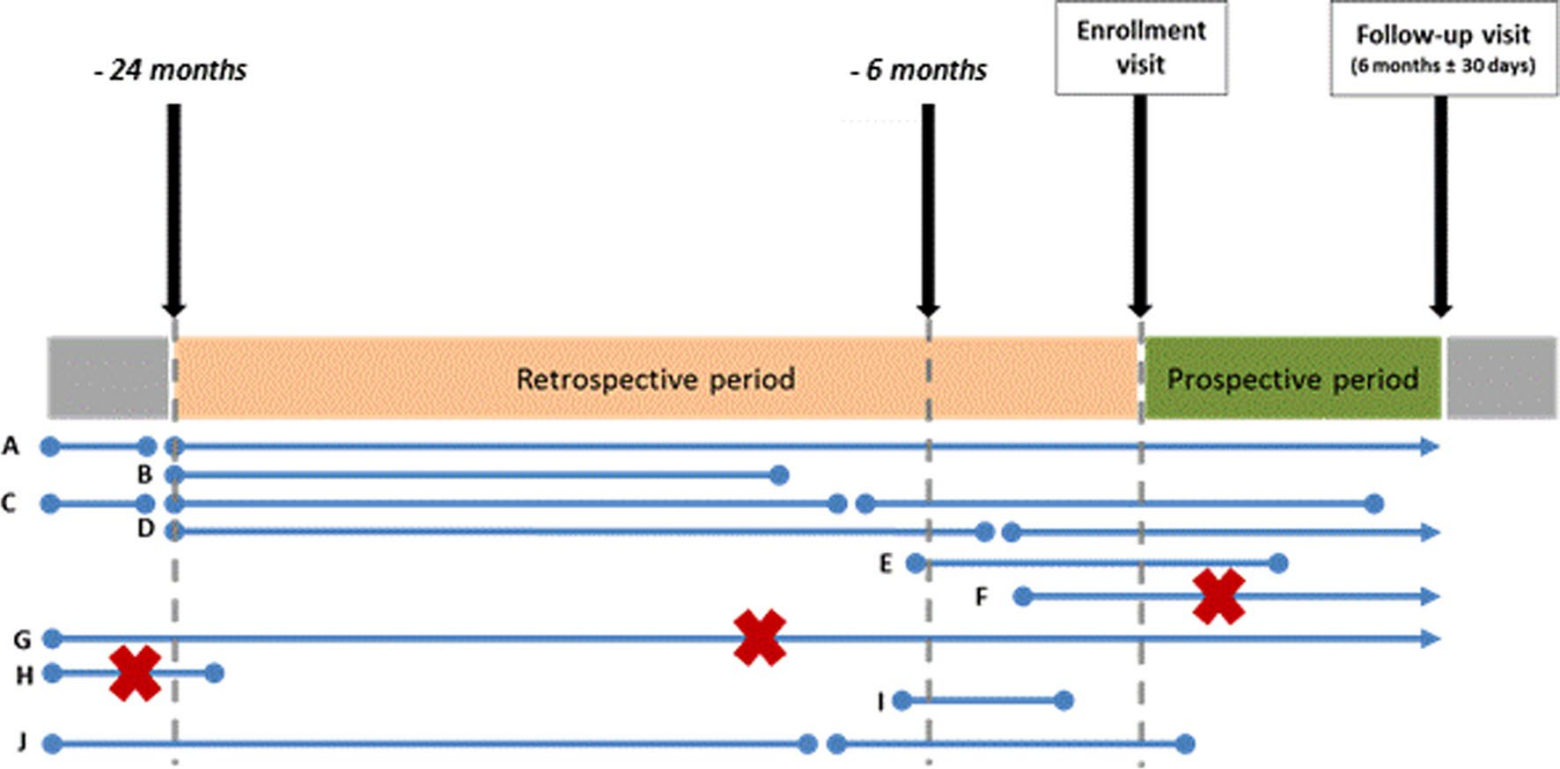
Patient’s scheme. Each letter represents one patient, and each blue line represents a biologic therapy line; if the line ends with a circle, then the treatment line was interrupted, while if the line ends with an arrow, then the treatment line was not interrupted. All patients from “**A**” to “**E**” were eligible for the study because in all these cases at least one line of biological therapy for psoriatic arthritis was initiated at least 6 months before enrolment visit but no more than 24 months before enrolment visit; also patient “**B**” was eligible, even though he/she has interrupted the treatment line before enrollment. Regarding patients “**A**” and “**C**”, only the biologic therapy lines initiated within the retrospective period were considered (e.g. for patient “**C**”, only 2nd and 3rd line was evaluated). Patients with a red cross (i.e. “**F**”, “**G**” and “**H**”) were not eligible for the study because in all these cases every line of biological therapy for psoriatic arthritis was initiated less than 6 months before enrolment visit (patient “**F**”) or more than 24 months before enrolment visit (patients “**G**” and “**H**”). Patient “**I**” was eligible for the study because in this case at least one line of biological therapy for psoriatic arthritis was initiated at least 6 months but no more than 24 months before enrolment visit. Patient “**J**” was eligible too, but only the 2nd therapy line was considered (because the first therapy line did not start within the retrospective period)

CHRONOS (EffeCtiveness of biologic treatments for psoriatic artHRitis in Italy: An ObservatioNal lOngitudinal Study of real-life clinical practice) was a multicenter, non-interventional study involving both retrospective and prospective data. The study was conducted in 20 Italian hospital rheumatology clinics. Patients were enrolled from September 2018 until September 2019 and data were collected until April 2020. Eligible patients satisfying inclusion criteria and not violating exclusion criteria were aged ≥ 18 years with diagnosis of PsA according to the treating rheumatologist, had initiated a biologic treatment more than 24 weeks and less than 24 months before enrolment visit (see Fig. 1) and had available data for DAS28 in the retrospective. Pregnant or breast-feeding women, receiving or having received biologic treatments as part of a clinical trial were excluded; treatment interruption before enrolment was not an exclusion criterion. Patients without available data of the clinical response at the start and at 6 months/1 year after treatment initiation were excluded from the evaluation of the primary objective.

Patients were withdrawn from the study in case of withdrawal of informed consent and privacy form, death, loss to follow-up, inclusion in a clinical trial involving treatment with biologic agents for PsA, or pregnancy. Stopping biologic treatment was not a reason for study exit.

At the enrolment visit, data since initiation of the earliest biologic were retrospectively collected from hospital medical charts or other clinical documents, while the minimum prospective observational period was 6 months (± 1 month), so that each patient was planned by design to have a total of observational period of at least 12 months except in case of early withdrawal (see Fig. 1). Patients who withdrew from the study were included in the analyses as long as they had available clinical outcomes.

### Outcome measures

The primary outcome of the study was the proportion of patients with PsA achieving clinical response by the EULAR DAS28 response criteria [18] to the biologic therapy initiated in the retrospective period which started most recently with respect to enrolment (henceforth referred to as “biologic treatment under analysis”); response was evaluated at 6 months and 1 year after the baseline (start of biologic treatment under analysis).

DAS28 has been successfully used for PsA [18, 19] and is frequently adopted in the clinical practice in Italy [20, 21]; it takes into account a 28 tender joint count (range 0–28), a 28 swollen joint count (range 0–28), erythrocyte sedimentation rate (ESR) or C-reactive protein (CRP), and patients’ general health (GH) measured by a visual analogue scale [22]. As EULAR suggests that DAS28 calculation may be based on ESR (hereafter named DAS28 ESR) or on CRP (hereafter named DAS28 CRP), both measurements were calculated in our study.

Moreover, as sensitivity analysis, the proportion of patients achieving response at 6 months and 1 year after treatment initiation was calculated according to the American College of Rheumatology (ACR) criteria, when available in the medical charts, in order to allow a comparison of the CHRONOS results with data from the literature. The ACR criteria have been widely used in clinical trials to measure the improvement induced by investigational treatments for rheumatoid arthritis and PsA [16, 23–27]. As they are not as commonly used in clinical practice in Italy, we did not select it as primary outcome. ACR20 responders should achieve a 20% improvement in tender or swollen joint counts as well as a 20% improvement in at least three out of the other five criteria (patient assessment, physician assessment, pain scale, disability/functional questionnaire, ESR or CRP). ACR50 and ACR70 follow similar patterns of definition.

The clinical response was evaluated also in terms of presence of dactylitis (number of patients with dactylitis and of affected fingers according to the judgment of the treating rheumatologist), enthesitis (presence and location evaluated by Leed Enthesitis Index, LEI), and presence of axial arthritis (according to the judgment of the treating rheumatologist) over the study observation period.

Secondary outcome measures were the Psoriasis Area and Severity Index (PASI) score—ranging 0–72 and increasing with increasing severity—, the Health Assessment Questionnaire Disability Index (HAQ-DI)—ranging 0–3 and increasing with increasing disability [28]—, and the Treatment Satisfaction Questionnaire for Medication-9 (TSQM-9) subscale scores—ranging from 0 to 100 with higher scores representing higher satisfaction [29, 30]. Switchers were defined as patients switching from a branded/biosimilar to a branded/biosimilar of another class (changes in dosage or frequency within the same therapy class were not considered as a switch); patients who discontinued stopped the biologic treatment under analysis before end of observation.

### Sample size

Enrolling 400 patients with PsA, 15% of whom might not be evaluable for the primary analysis, was considered feasible and hence the achievable precision for 340 patients was calculated for the primary endpoint at 6 months and 1 year after initiation of the biologic treatment under analysis. Based on the existing literature [31–38], an expected proportion of patients achieving response between 38.0 and 57.8% at 6 months and between 42.0 and 53.0% at 1 year was considered. The achieved precision was evaluated in terms of relative error (i.e. the ratio between 95% CI half-width of the expected proportion and the expected proportion itself) and it was considered good because it was lower than 15%.

### Statistical analysis

No formal statistical hypotheses were set. Descriptive analyses were performed; quantitative variables were described by mean, standard deviation, median, 25th and 75th percentile, minimum and maximum, while qualitative variables by absolute and relative frequency. Bilateral 95% confidence intervals were given where relevant.

Enrolled patients who did not meet the study criteria, were excluded from analyses. However, patients with follow-up visits performed outside the visit’s window defined by study protocol, were not excluded from analyses.

The patients with and without available response at 6 months and 1 year were compared in relation to the main patient characteristics at the start of biologic treatment (age, gender, ethnicity, duration of psoriasis and PsA, type of biologic treatment, number of prior biologic therapies and number of biologic therapies during the study) in order to evaluate possible selection bias and to better contextualize obtained results. *T*-test for normally distributed variables, non-parametric Wilcoxon Rank-Sum Test for non-normally distributed numerical variables and Chi-square or Fisher test for categorical variables were performed to compare patients with vs without available outcome measures.

The proportion of eligible patients discontinuing or switching was calculated and a Kaplan–Meier analysis was done in order to evaluate the persistence from the biologic treatment under analysis. The persistence was defined as months of treatment and the event was defined as discontinuation of the treatment or switch to another one. Dropped-out patients who did not have the event before discontinuation or patients who had no event at the end of observation period were censored at the date of the drop out or of the last available visit, respectively.

Site monitoring, data management, and statistical analysis were performed by MediNeos (Modena, Italy). Database management and data analysis were performed using SAS Enterprise Guide v. 7.1 and SAS 9.4.

## Results

### Patient population

**Fig. 2:**
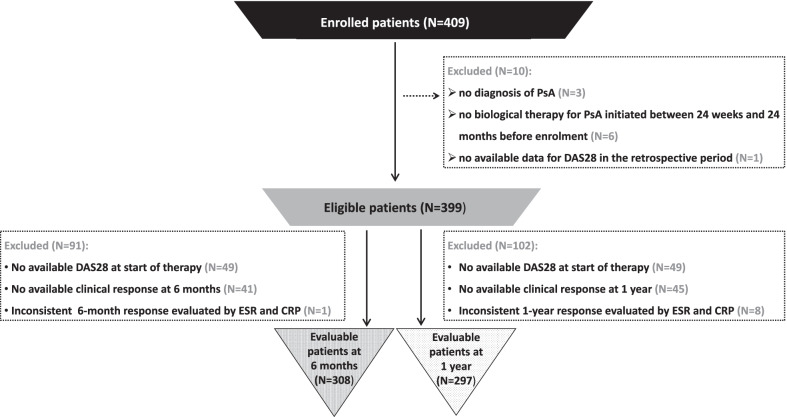
Patient disposition

A total of 409 patients were enrolled; among these, the eligible patients were 399 (97.6%) because 10 patients did not meet the inclusion criteria (3 patients had no diagnosis of PsA, 6 patients had no biological therapy for PsA initiated between 24 weeks and 24 months before enrolment and 1 patient had no available data for DAS28 in the retrospective period) (see Fig. 2). Seventeen patients (4.3% of the eligible) prematurely discontinued the study (11 were lost to follow-up, 2 became pregnant, 2 due to Covid-19 emergency, 1 died and 1 moved to another structure). Not all eligible patients were evaluable for the primary analysis on clinical response (based on DAS28) at the study time point; as 91 patients at 6 months and 102 patients at 1 year did not have available data on clinical response, 308 and 297 patients were evaluable at 6 months and 1 year, respectively (see Fig. 2).

Notable differences between patients with vs without available response were observed only in relation to the duration of PsA with a median (25th–75th percentile) time of 5.0 (2.1–10.8) years versus 3.5 (2.2–6.3) years in patients with vs without available response at 1 year, respectively (Wilcoxon Rank-Sum Test *p*-value = 0.03) and in the number of received biologic therapies during the study (85.5%, 9.8%, 4.0%, 0.7% of patients with available 1-year response vs. 67.6%, 27.5%, 3.9%, 1.0% of patients without available response received 1, 2, 3 or 4 biologics, respectively; Fisher exact Test *p*-value = 0.0002).

The mean observation period in all eligible patients was 20.3 months (SD 5.7). Of the 399 eligible patients, 172 (43.1%) were males and 227 (56.9%) were females. The mean age was 52.4 years (SD 11.6). Overall, 61.5% of patients were overweight (36.7%) or obese (24.8%). The mean duration of psoriasis since first diagnosis (N = 226) was 15.3 years (SD 12.2), whereas the mean duration of PsA (N = 392) was 7.2 years (SD 6.9). The socio-demographic and main clinical characteristics are presented in Table 1.

**Table 1:** Socio-demographic and main clinical characteristics at enrolment

Age at enrolment, years	Mean (SD)	52.4 (11.6)
Males	N (%)	172 (43.1%)
Caucasian	N (%)	398 (99.7%)
*Smoking status at enrolment*		
Non-smoker	N (%)	236 (67.8%)
Current smoker	N (%)	63 (18.1%)
Previous smoker	N (%)	49 (14.1%)
UNK	N	51
BMI classes at enrolment*		
Underweight	N (%)	9 (2.9%)
Normal weight	N (%)	111 (35.7%)
Overweight	N (%)	114 (36.7%)
Obese	N (%)	77 (24.8%)
UNK	N	88
Duration of psoriasis at start of biologic treatment under analysis, years (N = 226)	Mean (SD)	15.3 (12.2)
Duration of PsA at start of biologic treatment under analysis, years (N = 392)	Mean (SD)	7.2 (6.9)
DAS28 ESR at start of biologic treatment under analysis (N = 279)	Mean (SD)	4.0 (1.3)
DAS28 CRP at start of biologic treatment under analysis (N = 312)	Mean (SD)	3.8 (1.2)

UNK: Unknown. *Underweight: BMI < 18.5, normal weight: BMI 18.5–24.9, overweight: BMI 25–29.9, obese: BMI ≥ 30

Percentages and descriptives calculated over the total number of eligible patients (N = 399), if not otherwise specified

Regarding type of PsA, 44.6% (N = 178) of patients had symmetric polyarthritis, 38.8% (N = 155) asymmetric oligoarthritis, 20.3% (N = 81) spondylitis, 4.8% (N = 19) predominant distal interphalangeal arthritis, and 0.8% (N = 3) arthritis mutilans. Among eligible patients, 60.9% (N = 243) had comorbidities at enrollment, the most common being hypertension (N = 127, 31.8%), followed by diabetes (N = 38, 9.5%) and hypercholesterolemia/dyslipidemia (N = 37, 9.3%). Other reported comorbidities were: thyroid diseases (N = 32, 8.0%), obesity (N = 25, 6.3%), autoimmune diseases (N = 21, 5.3%), osteoporosis (N = 20, 5.0%), fibromyalgia (N = 16, 4.0%), depression (N = 15, 3.8%), asthma (N = 12, 3.0%), hepatitis (N = 9, 2.3%).

In the eligible population, the most commonly used biologic treatment under analysis was secukinumab (40.4%; N = 161), followed by adalimumab (biosimilars included, 17.8%; N = 71), and etanercept (biosimilars included, 16.5%; N = 66). Other biologic treatments under analysis were: certolizumab (9.8%; N = 39), ustekinumab (7.5%; N = 30), golimumab (5.0%; N = 20) and infliximab (biosimilars included, 3.0%; N = 12). TNF-inhibitors were used by 52.1%; (N = 208) of the patients.

While 46.6% (N = 186) of the patients were naïve to biologics, 33.3% (N = 133), 10.0% (N = 40), 6.8% (N = 27) and 3.3% (N = 13) had received one, two, three, four or more lines, respectively. The total mean duration of the biologic treatment under analysis was 18.6 (SD 6.5) months; overall, 97.7% (N = 390) had been on treatment for ≥ 6 months, whereas the median duration of exposure to any biologics since the diagnosis of PsA until the end of observation was 23.9 (25th–75th percentile: 17.2–37.9) months.

In patients on secukinumab 42.9% were naïve to biologics, 31.1% had received 1 previous biologic line and 26.1% received ≥ 2 previous biologic lines; in patients treated with TNFi 51.9% were naïve to biologics, while 33.2% and 14.9% had received 1 or ≥ 2 previous biologic lines, respectively.

Other factors as age, gender, ethnicity, smoking status, BMI, comorbidities, PsA manifestation, time since PsA and Pso diagnosis, months of exposure to biologic therapy under analysis, number of lines received during study, DAS28 at start of biologic therapy under analysis were largely similar across the treatment groups (see Additional file 1).

Concomitant topical treatments were used for psoriasis by 8.0% of study patients (N = 32), mainly topical corticosteroids (6.0%, N = 24) or vitamin D analogues (3.5%, N = 14). One patient received phototherapy. Systemic pharmacological treatments for PsA or psoriasis other than biologics were received by 46.6% (N = 186), mainly methotrexate (28.1%), non-steroidal anti-inflammatory drugs (NSAIDs, 12.3%), and systemic corticosteroids, either orally or parenterally (10.8%) (Table 2).

Rehabilitation therapy was received by 5 patients (1.3%), either physiotherapy (N = 4) or kinesiotherapy (N = 1).

**Table 2:** Systemic pharmacological treatments other than biologicals assumed during the study observation period for psoriatic arthritis or psoriasis

	N (%)
Patients receiving other pharmacological therapies for psoriatic arthritis or psoriasis during the observation period	186 (46.6%)
Methotrexate	112 (28.1%)
NSAIDs	49 (12.3%)
Systemic corticosteroids (oral or parenteral)	43 (10.8%)
Leflunomide	18 (4.5%)
Sulfasalazine	15 (3.8%)
Cyclosporine	7 (1.8%)
Apremilast	4 (1.0%)
Azathioprine	1 (0.3%)

NSAIDs: non-steroidal anti-inflammatory drugs

Percentages calculated over the total number of eligible patients (N = 399)

### Primary objective: effectiveness

**Fig. 3:**
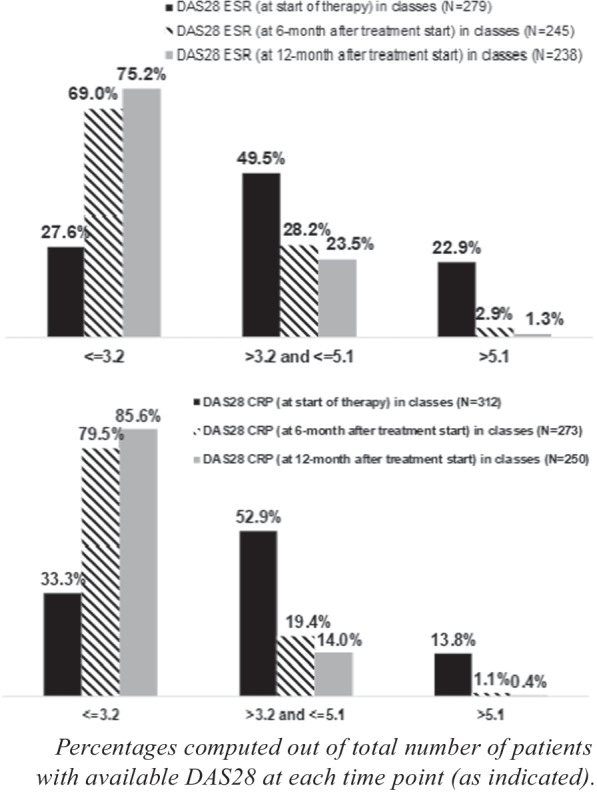
Distribution of patients by DAS28 ESR and DAS28 CRP classes at start of biologic treatment under analysis, and after 6 months and 1 year after treatment start

The proportion of patients with DAS28 ESR or DAS28 CRP > 3.2 decreases after 6 months and 1 year of treatment (Fig. 3).

Among the evaluable patients, the proportion of overall responders according to EULAR DAS28 criteria was 71.8% (95% CI: 66.7–76.8%) at 6 months and 68.0% (95% CI: 62.7–73.3%) at 1 year. The proportions of DAS28 responders at 6 months and 1 year for the secukinumab-treated and the TNFi-treated patients were 73.4% (95% CI: 65.8–81.1%). 69.6% (95% CI: 61.5–77.7%), and 71.9% (95% CI: 64.9–78.8%), 70.5% (95% CI: 63.1–77.8%) respectively.

In the overall population, ACR20, ACR50 and ACR70 responder rates were 41.2%, 29.4%, 17.1% at 6 months, and 34.9%, 26.7%, 18.4%, at 1 year, respectively. The response rates by treatment subgroups are detailed in Table 3.

**Table 3:** Proportion of patients achieving clinical response at 6 months and 1 year (according to ACR20, ACR50 and ACR70 criteria)

	Overall	Secukinumab	TNFis
	n (%)	n (%)	n (%)
6 months			
ACR20 response	n = 194	n = 75	n = 105
	80 (41.2%)	34 (45.3%)	41 (39.0%)
ACR50 response	n = 194	n = 75	n = 105
	57 (29.4%)	25 (33.3%)	28 (26.7%)
ACR70 response	n = 199	n = 77	n = 108
	34 (17.1%)	14 (18.2%)	18 (16.7%)
12 months			
ACR20 response	n = 189	n = 74	n = 100
	66 (34.9%)	26 (35.1%)	36 (36.0%)
ACR50 response	n = 191	n = 75	n = 101
	51 (26.7%)	22 (29.3%)	26 (25.7%)
ACR70 response	n = 196	n = 76	n = 105
	36 (18.4%)	13 (17.1%)	21 (20.0%)

Percentages calculated over the total number of patients with available evaluation of ACR at each time point (n is indicated in the table)

The number of patients with dactylitis decreased from 35 at start of therapy (23.6% out of 148 patients with available evaluation) to 5 already at 6 months (3.5% out of 142 patients with available evaluation; details in Table 4).

**Table 4:** Patients with dactylitis and enthesitis at start of biologic treatment under analysis, and after 6 months and 1 year

	Start of therapy	At 6 months	At 1 year
	n (%)	n (%)	n (%)
Dactylitis	N = 148	N = 142	N = 134
Patients with dactylitis	35 (23.6%)	5 (3.5%)	4 (3.0%)
Patients with 1 finger with dactylitis	24 (16.2%)	2 (1.4%)	4 (3.0%)
Patients with 2 fingers with dactylitis	8 (5.4%)	2 (1.4%)	0
Patients with ≥ 3 fingers with dactylitis	3 (2.0%)	1 (0.7%)	0
Leeds Enthesitis Index (LEI)	N = 147	N = 133	N = 132
None	98 (66.7%)	121 (91.0%)	120 (90.9%)
Lateral epicondyles of the humerus R	22 (15.0%)	4 (3.0%)	2 (1.5%)
Lateral epicondyles of the humerus L	19 (12.9%)	7 (5.3%)	4 (3.0%)
Achilles tendon insertion R	20 (13.6%)	5 (3.8%)	3 (2.3%)
Achilles tendon insertion L	19 (12.9%)	6 (4.5%)	4 (3.0%)
Medial femoral condyles R	9 (6.1%)	4 (3.0%)	0 (0.0%)
Medial femoral condyles L	9 (6.1%)	4 (3.0%)	5 (3.8%)

Percentages calculated over the number of patients specified in the column headings with available evaluation of dactylitis (or enthesitis) at each time point. Patients with presence of enthesitis could specify multiple sites. R: right; L: left

Overall patients with enthesitis were 33.3% at start of therapy (N = 49 out of 147 patients with available evaluation of enthesitis) and decreased to 9.0% at 6 months (N = 12 out of 133 patients with available evaluation of enthesitis). Details on the progress of enthesitis at the different locations are reported in Table 4. Patients with evaluation of axial arthritis were only 102 at baseline, 82 at 6 months and 76 at 1 year. Among those, 56.9% had axial arthritis at start of therapy (N = 58 out of 102), 48.8% at 6 months (N = 40 out of 82) and 46.1% at 1 year (N = 35 out of 76).

### Secondary objectives

In terms of extent and severity of psoriasis, the mean (SD) PASI at start of therapy, after 6 months and 1 year in the overall eligible patients with available score at time points (N = 87) were 3.2 (5.1), 0.6 (1.3) and 0.6 (1.3), respectively. The PASI decreased on average (SD) between the start of therapy and the 6-month assessment of 2.7 (4.6) points and of 2.6 (4.7) points between the start of therapy and 1 year.

Patients’ functional status was of mild-moderate disability at treatment start. The mean (SD) HAQ-DI in the overall eligible population with available HAQ-DI at all timepoints (N = 65) was 0.9 (0.6) at treatment start, and 0.7 (0.6) at 6 and 12 months. The mean (SD) decrease in HAQ-DI between the start of therapy and the 6-month assessment was 0.2 (0.6) and remained substantially the same between the start of therapy and 1 year (mean (SD) decrease: 0.2 (0.7)).

Lastly, the TSQM-9 subdomain assessment showed mean scores at enrolment visit (N = 396) of 66.1 (SD 21.9) for effectiveness, 77.0 (16.0) for convenience, and 66.7 (18.4) for global satisfaction and slightly increased at the 6-month follow-up visit (N = 365) to 69.2 (20.3), 78.3 (15.0), and 67.9 (18.4), respectively.

**Fig. 4:**
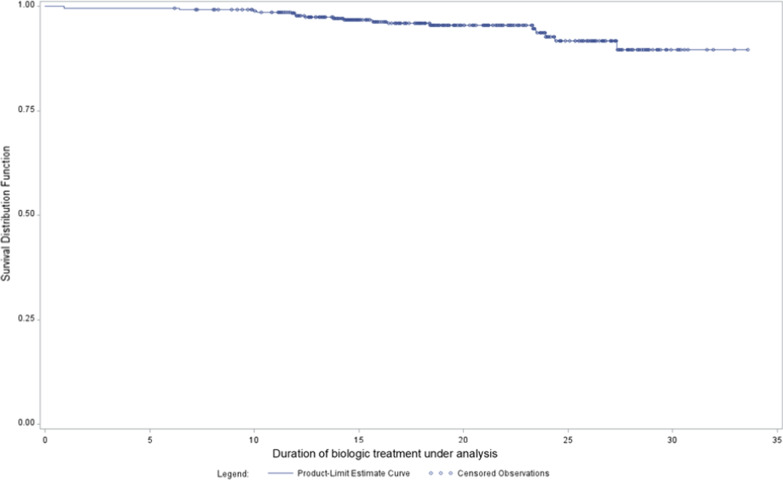
Persistence of the biologic treatment under analysis: Kaplan–Meier survival curve

Only 19 patients out of 399 (4.8%) discontinued the biologic during the study and 33 (8.3%) switched to a different biological. The high persistence on biologic treatment was confirmed by the Kaplan–Meier analysis, showing 6-month and 1-year probabilities of treatment discontinuation/switch of 3% and 7%, respectively (Kaplan–Meier survival curve is shown in Fig. 4).

## Discussion

Our study was designed with the aim of providing real world data on the effectiveness of biologics in the treatment of PsA in the Italian clinical practice. In our PsA population, almost all patients were or had been on the analyzed biologic treatment for at least 6 months, with an average duration of treatment of over one and a half years. Overall, the mean duration of any biologic treatment since PsA diagnosis was over 2 years. For almost half of our patients, the biologic treatment under analysis was the first biologic medication assumed. Other systemic treatments for PsA were taken by almost half of patients (46.6%) and consisted mainly in methotrexate (28.1%), whose possible association with biologicals is actually foreseen in the indications of many biological medications.

Regarding the primary objective, unfortunately we had almost 20% fewer evaluable patients among the eligible ones, due to lack of available information about the DAS28 response. The analysis performed to highlight any differences between patients with and without available clinical response gave somewhat contradictory information: patients with available data had higher duration of PsA but had received fewer biologic therapies during study, which does not allow to establish whether they were more or less likely to respond to the biological under analysis. The overall EULAR DAS28 responder rate at 6 months was rather high (71.8%). Between-treatment comparisons were not in the scope of our study; however, given that most of the patients were treated with secukinumab or TNFis, we also looked separately into the results of these two major treatment subgroups. A similar response was observed in both treatment subgroups at 6 months (73.4% with secukinumab, 71.9% with TNFis). At 1 year, the overall responder rate was a little lower (68.0%), similar in both treatment subgroups (69.6% with secukinumab, 70.5% with TNFis). Given that TNFis are usually used earlier in the biological treatments’ progression in the Italian clinical practice, to better characterize the two larger treatment subgroups in our study, we checked whether there were some differences in the number of biological lines assumed before study treatments in the two main treatment subgroups; even if no statistical differences were observed between treatment groups, there are more bio-naive patients in TNFi group and this may have influenced the effectiveness since bio-naive patients respond better than bio-experienced patients (see Additional file 1).

Considering ACR criteria, responders were somewhat more with secukinumab at 6 months but tended to be lower at 1 year. The ACR response rates allow us to better compare our real-life data with those of the literature. For example, in the secukinumab FUTURE 1 and 2 pivotal trials in PsA [39, 40], ACR20 response rates at 6 months were between 50 and 54% and were maintained through 1 year. In our study, response rates are lower. What was more striking, is that our ACR response rates, unlike in RCTs, showed to lower over time, even from 6 months to 1 year. Also looking at the pivotal RCTs of the other most frequently used biological treatments in our study, i.e. adalimumab [41] and etanercept [42, 43], ACR responses in RCTs are more or less higher than in the CHRONOS study and again are reported, at least up to 1 year, to be maintained. Typically, patients and the course of treatment in the daily clinical practice are different from RCTs: real world studies include different patient population than RCTs, less selected, less homogeneous, often with more comorbidities and previously exposed to different biologic treatments [44, 45]. Furthermore, data from registries have suggested that biological agents’ doses are generally lower than those indicated in the drug labels [46]. Moreover, overweight and obese patients were highly represented in our cohort and it has been reported that obesity may hamper the effect of some biologic agents in axial spondyloarthritis and PsA [47]. Looking at the specific clinical features of PsA, treatment with biologicals in our patients showed to reduce dactylitis and enthesitis rapidly and dramatically. A modest reduction in the proportion of patients with axial arthritis was observed during the study. According to clinical practice, evaluation of axial arthritis was performed in a small proportion of patients (26% and < 20% of eligible patients had available evaluation at start of therapy and at 1 year, respectively). Also, dactylitis and enthesitis seem not to be routinely performed (34% and 37% of eligible patients had available evaluation at start of therapy and at 1 year, respectively). Therefore, the reliability of these positive results, especially those about axial arthritis, is somewhat impaired due to the limited and decreasing number of patients with available data.

The skin burden of psoriasis was low at start of therapy, suggesting that psoriasis was already under a rather good control in these PsA patients. Nevertheless, the mean PASI decreased after 6 months showing an improving of the skin disease. At start of therapy, our patients had a mild disability that improved slightly during biological treatment, likely due to the already low initial level of impairment. Treatment withdrawals and switches were few, confirming the high persistence with biological treatments reported in the literature [48–51]. Consistently, patients’ satisfaction with the study treatment was rather high for all three TSQM-9 domains.

The limitations of the CHRONOS are mainly due to its observational nature. Regarding potential confounders, the population could be in some way heterogeneous, in terms of clinical history, previous treatments for PsA, durations of observation and number of biologic lines. Moreover, there might have been some differences in patients’ characteristics by treatment group (as higher number of previous biologic therapies), somehow affecting results of the stratified response analyses; however, main potential confounding factors were evaluated and resulted well balanced by treatment group (see Additional file 1). Another limitation may consist in a certain inhomogeneity in the quality of the collected data since the study included both a retrospective and a prospective period. Furthermore, since biologic medications were generally injected at home, it is possible that in certain cases patients were not fully compliant to the prescribed regimen (in terms of posology, frequency of administration, stopping rules, etc.), which may have affected somewhat their effectiveness, especially in the longer term. In addition, not all enrolled patients had available complete data on clinical response at all time points. In order to manage this potential selection bias, as already discussed, patients with and without available response at 6 months and 1 year were compared in relation to the main patient characteristics at the start of biologic treatment. One of the actions put in place in order to reduce the impact of such missing data on study objectives evaluation was the enlargement of the tolerability windows of the main outcomes during data analysis, considering as valid from a clinical point of view also data collected few months before or after the planned time points of interest. This action allowed to reach a total number of patients evaluable for the primary objective at 6 months of 308, about 9% lower than the planned sample size but, as already pointed out, allowing precise estimates of the primary endpoints.

## Conclusions

In conclusion, the CHRONOS study provides real-world data on the effectiveness of biologics in PsA in the Italian rheumatological daily practice. Our results confirm the efficacy of biologic therapy reported in RCTs across various outcome measures, including patients’ satisfaction, although to a generally lower extent which might be due some of the study limitations. Persistence on biologic treatment up to 1 year was high with low probabilities of treatment discontinuation or switch.

## Supplementary Information

**Additional file 1**: Socio-demographic and clinical characteristics at enrollment/start of biologic treatment under analysis (in Secukinumab and TNFis patients). In this file main socio-demographic and clinical characteristics at enrollment/start of biologic treatment under analysis are described in the groups of patients treated with Secukinumab and with TNFis.

## Data Availability

The data that support the findings of this study are available from authors and Novartis Farma S.p.A. but restrictions apply to the availability of these data, which were used under license for the current study, and so are not publicly available. Data are however available from the authors upon reasonable request and with permission of the authors and Novartis Farma S.p.A.

## Abbreviations

ACR: American College of Rheumatology
CRP: C-reactive protein
DAS28: Disease activity score 28 joints
DMARDs: Disease modifying anti-rheumatic drugs
ESR: Erythrocyte sedimentation rate
EULAR: European league against rheumatism
GH: General health
HAQ-DI: Health Assessment Questionnaire Disability Index
IL: Interleukin
LEI: Leed Enthesitis Index
PASI: Psoriasis Area and Severity Index
PsA: Psoriatic arthritis
RCTs: Randomized clinical trials
RWE: Real-world evidence
TNFis: TNF inhibitors
TSQM-9: Treatment satisfaction questionnaire for medication-9
