# Supplementary material for: Real-world evidence of biologic treatments in psoriatic arthritis in Italy: results of the CHRONOS (EffeCtiveness of biologic treatments for psoriatic artHRitis in Italy: an ObservatioNal lOngitudinal Study of real-life clinical practice) observational longitudinal study
Source: BMC Rheumatol. 2022 Sep 12;6:57. doi: 10.1186/s41927-022-00284-w (PMC9464489; doi:10.1186/s41927-022-00284-w)
Supplement: Supplementary file 1 — Additional file 1: Socio-demographic and clinical characteristics at enrollment/start of biologic treatment under analysis (in Secukinumab and TNFis patients). In this file main socio-demographic and clinical characteristics at enrollment/start of biologic treatment under analysis are described in the groups of patients treated with Secukinumab and with TNFis. [file 41927_2022_284_MOESM1_ESM.docx]

### Additional file 1.

**Socio-demographic and clinical characteristics at enrollment/start of biologic treatment under analysis (in Secukinumab and TNFis patients)**

|  | **Secukinumab** | **TNFis** |
| --- | --- | --- |
| Age at enrolment (years), mean (SD) | 53.4 (11.2) | 51.6 (11.6) |
| Males, N (%) | 70 (43.5) | 90 (43.3) |
| Caucasian, N (%) | 161 (100.0) | 208 (100.0) |
| Smoking status at enrolment*, N (%) | |  |
| Non-smoker | 98 (69.0) | 121 (67.6) |
| Current smoker | 27 (19.0) | 30 (16.8) |
| Previous smoker | 17 (12.0) | 28 (15.6) |
| UNK | 19 | 29 |
| BMI classes at enrolment*, N (%) |  |  |
| Underweight (BMI < 18.5) | 3 (2.5) | 6 (3.7) |
| Normal weight (BMI 18.5-24.9) | 45 (37.2) | 57 (34.8) |
| Overweight (BMI 25-29.9) | 43 (35.5) | 62 (37.8) |
| Obese (BMI ≥ 30) | 30 (24.8) | 39 (23.8) |
| UNK | 40 | 44 |
| Duration of psoriasis at start of biologic treatment under analysis (years), median (25^th^-75^th^ percentile) | N=98  9.4 (3.7-22.4) | N=107  13.8 (5.7-21.7) |
| Duration of PsA at start of biologic treatment under analysis (years), median (25^th^-75^th^ percentile) | N=159  4.5 (2.2-9.2) | N=204  4.2 (2.1-10.5) |
| DAS28 ESR at start of biologic treatment under analysis, mean (SD) | N=128  4.1 (1.4) | N=136  3.9 (1.3) |
| DAS28 CRP at start of biologic treatment under analysis, mean (SD) | N=134  3.9 (1.1) | N=153  3.7 (1.2) |
| Total duration of biologic treatment under analysis (months), mean (SD) | 18.8 (6.6) | 18.7 (6.6) |
| N of biologic therapies before biologic treatment under analysis**, N (%) |  |  |
| 0 (naïve patients) | 69 (42.9) | 108 (51.9) |
| 1 | 50 (31.1) | 69 (33.2) |
| 2 | 19 (11.8) | 16 (7.7) |
| 3 | 14 (8.7) | 11 (5.3) |
| >=4 | 9 (5.6) | 4 (1.9) |
| N of biologic therapies received during study, N (%) |  |  |
| 1 | 130 (80.7) | 171 (82.2) |
| 2 | 24 (14.9) | 27 (13.0) |
| 3 | 5 (3.1) | 9 (4.3) |
| 4 | 2 (1.2) | 1 (0.5) |
| Comorbidities at start of biologic treatment under analysis, N (%) | 99 (61.5) | 123 (59.4) |
| Hypertension | 51 (31.7) | 64 (30.9) |
| Diabetes | 15 (9.3) | 20 (9.7) |
| Hypercholesterolemia/dyslipidemia | 13 (8.1) | 19 (9.2) |
| Thyroid diseases | 13 (8.1) | 8 (3.9) |
| Type of PsA, N (%) |  |  |
| symmetric polyarthritis | 79 (49.1) | 86 (41.3) |
| asymmetric oligoarthritis | 57 (35.4) | 83 (39.9) |
| spondylitis | 33 (20.5) | 43 (20.7) |
| predominant distal interphalangeal arthritis | 6 (3.7) | 12 (5.8) |
| arthritis mutilans | 2 (1.2) | 1 (0.5) |

Percentages and descriptives calculated over the total number of eligible patients treated with Secukinumab (N=161) or TNFis (N=208), if not otherwise specified.

*Percentages computed on available responses.

UNK: Unknown.
